# Management of Recurrent Endometrial Cancer or Atypical Endometrial Hyperplasia Patients After Primary Fertility-Sparing Therapy

**DOI:** 10.3389/fonc.2021.738370

**Published:** 2021-09-09

**Authors:** Junyu Chen, Dongyan Cao, Jiaxin Yang, Mei Yu, Huimei Zhou, Ninghai Cheng, Jinhui Wang, Ying Zhang, Peng Peng, Keng Shen

**Affiliations:** ^1^Department of Obstetrics and Gynecology, Peking Union Medical College Hospital, Chinese Academy of Medical Science & Peking Union Medical College, Beijing, China; ^2^National Clinical Research Center for Obstetric & Gynecologic Diseases, Beijing, China

**Keywords:** endometrial cancer, atypical endometrial hyperplasia, recurrence, fertility-sparing, re-treatment

## Abstract

**Objective:**

To evaluate the efficacy and prognosis of fertility-sparing re-treatment on patients with recurrent endometrial cancer (EC) and atypical endometrial hyperplasia (AEH) who wish to preserve their uterus after complete remission (CR) for primary conservative therapy.

**Methods:**

We performed a retrospective study on recurrent EC or AEH patients who received fertility-sparing re-treatment after achieving CR. Data regarding clinicopathological factors, adverse events, treatment efficacy, tumor prognosis, and reproductive outcome were analyzed.

**Results:**

Of the 98 recurrent patients with a median disease-free interval period of 19 (3–96) months, 18 patients decided to receive hysterectomy directly, and 80 patients received fertility-preserving re-treatment. Seventy-one (88.6%) cases achieved CR, 96.0% in AEH and 75.8% in EC patients, with the 6 (3–16) months’ median CR time. Seven (8.8%) patients failed to achieve CR and then underwent the hysterectomy: one partial response (PR), four stable disease (SD), and two progressive disease (PD). Forty-nine women attempted to get pregnant after CR, 13 (26.5%) became pregnant, seven (14.3%) successfully delivered, and six (12.2%) miscarried. During the follow-up period, 22 (31.0%) women had developed a second relapse with the median recurrence time of 12 (4–90) months, and 10 patients decided to receive the third round of fertility-sparing treatment. Seven (70.0%) patients, 33.3% in EC and 85.7% in AEH, achieved CR again. Hysterectomy was performed in two (20.0%) patients due to SD. After the third-round treatment, six women had the desire to conceive but no one became pregnant successfully.

**Conclusion:**

For patients with recurrent EC and AEH after primary conservative treatment, fertility-preserving re-treatment can still achieve a promising response, and patients have possibilities of completing childbirth.

## Introduction

Endometrial cancer (EC) is one of the most common gynecologic cancer whose incidence is increasing rapidly in recent years (1). Conservative management for EC or atypical endometrial hyperplasia (AEH) patients who wish to preserve their fertility has been applied and showed encouraging treatment and reproductive outcomes (2–4). However, a significant portion of patients experience recurrence after achieving complete remission (CR) (5–7). Definitive surgery including hysterectomy with or without lymphadenectomy was recommended for recurrent patients. But some patients who have not had a successful pregnancy at the time of recurrence may still want to preserve their fertility. Considering most recurrent diseases in EC or AEH cases involve well-differentiated tumors confined to the endometrium, the second round of fertility-sparing therapy could be performed (8–10). However, little is known about the outcomes of the fertility-preserving re-treatment in recurrent cases, and few studies have addressed the efficacy of re-treatment for recurrent patients that have been published or only reported as a part of their wider analyses. Therefore, this study aimed to analyze the outcomes of fertility-sparing re-treatment in patients with recurrent EC or AEH.

## Method

### Patients Recruited

Recurrent disease was defined as the presence of EC or AEH in patients after achieving CR by primary fertility-preserving treatment (11). Patients with recurrent disease were included between January 2013 and June 2021 at the Department of Obstetrics and Gynecology, Peking Union Medical College Hospital (PUMCH).

Patients’ information was collected from medical records; and a prospectively maintained database, which represented a standard protocol, was followed for all patients. The inclusion criteria were as follows after systematic pre-treatment evaluation, which was similar to the primary treatment inclusion criteria: 1) women under 45 years old who desire to preserve their fertility; 2) histologically confirmed AEH or EC after CR; 3) Grades 1–2; 4) no signs of myometrial invasion or extrauterine metastasis by enhanced magnetic resonance imaging (MRI); 5) no contraindication of the drugs; 6) written informed consent obtained; and 7) patients were followed up regularly, with full text and complete data available. The diagnosis of the histological type was based on the endometrial curettage at the time of recurrence. This study was approved by the Ethics Committee of PUMCH.

### Treatment Methods

Two regimens were used: 1) progestin therapy: oral medroxyprogesterone acetate (MPA) 500 mg daily or megestrol acetate (MA) 160 mg b.i.d.; 2) gonadotropin-releasing hormone agonist (GnRHa)-based therapy: a combination of subcutaneous injection of 3.75 mg of GnRHa every 4 weeks and levonorgestrel-releasing intrauterine system (LNG-IUS) (Mirena; Bayer Health Care Pharmaceutical Inc.) insertion constantly/oral aromatase inhibitor (AI) (letrozole) 2.5 mg daily. The distribution of the patients to these two regimens was made based on the initial regimen, physicians’ recommendation, and patients’ choices. GnRHa-based regimen was recommended for patients who were contraindicated or unsuitable for oral progestin such as body mass index (BMI) ≥28, level of alanine aminotransferase (ALT) ≥1.5 normal value, or no response to previous standard high-dose oral progestin regimen. During the process of treatment, weight loss plans including diet control and exercise recommendation were provided to all patients. Outpatient visits were arranged every 1–2 months during the treatment; symptoms such as vaginal spotting and abdominal pain were recorded; and physical examination including body weight and lab tests including complete blood counts and biochemistry panels were performed. A trans-vaginal ultrasound scan was performed at every visit to assess the endometrium and exclude extrauterine abnormalities. Histological response was determined by endometrial curettage under hysteroscopic evaluation every 3–4 months (one course) during the treatment.

### Response Evaluation

Pathological response to treatment was categorized as complete response (CR), partial response (PR), stable disease (SD), and progressive disease (PD). CR was defined as the absence of evidence of hyperplasia or carcinoma. PR was defined as histological regression or endometrial decidual change. SD was defined as the persistence of disease as initially diagnosed. PD was defined as progression to a lesion of higher grade or PD including myometrial invasion, extrauterine disease, or lymph node metastasis. Patients with PR or SD continued the treatment for an additional one to two courses, whereas those with PD were immediately proposed to receive hysterectomy. Those who had the persistent or worsening disease after 12 months of therapy were considered to have failed and were also recommended surgery. Once achieved CR, patients who desire to get pregnant were encouraged to conceive or to undergo assisted reproductive technology (ART). Those with CR who had no birth plan temporary were prescribed oral contraceptives, low-dose cyclic progestin, or LNG-IUS insertion to prevent a recurrence.

### Follow-Up

After the documentation of CR, all patients were regularly followed up for a prolonged period with 3–6 months’ intervals. During each follow-up visit, the following information was collected: menstruation period or abnormal vaginal bleeding, results of trans-vaginal ultrasound scan or MRI if necessary, and data relating to the second relapse (interval between CR and recurrence, diagnosis of recurrence, treatment, and survival outcomes). Fertility outcomes including time of gestation, using ART, obstetrical complications, and delivery were also documented. If the patient received a hysterectomy, the reason and histological results of the surgery were also collected.

### Statistical Analyses

Statistical analysis was performed using IBM SPSS for Windows (version 22.0). Categorical variables are summarized in frequency tables, whereas continuous variables are presented as median (range). Frequency distributions were compared using chi-squared or Fisher’s exact tests, and median values were compared using Mann–Whitney U tests. For all statistical tests, the differences were considered statistically significant when p-values were <0.05.

## Results

### Characteristics of Patients With Recurrence

Totally, 98 patients relapsed with the median recurrence time of 19 (3–96) months after the primary conservative therapy. The clinical characteristics of patients with recurrence are shown in **Table 1**. Fifty-three (54.1%) patients were diagnosed as AEH, and 46 (45.9%) were diagnosed as EC at the first time of recurrence. The median age at first recurrence was 33 years, ranging from 16 to 47 years. Median BMI was 24.9 (16.7–41.1), and seven (7.1%) patients had co-occurrence of diabetes mellitus (DM). Sixty-two (63.2%) women were nulliparous, and 27 (27.5%) had comorbidity including polycystic ovary syndrome (PCOS) and/or endometriosis.

**Table 1:** Patient’s characteristics.

Characteristics	First recurrence (n = 98)	Second recurrence (n = 22)
Age (years), median (range)	33 (16–47)	36 (29–43)
BMI (kg/m^2^), median (range)	24.9 (16.7–41.1)	23.7 (16.6–34.9)
Histology		
EC	46 (45.9%)	10 (45.5%)
AEH	53 (54.1%)	12 (54.5%)
Comorbidity		
PCOS	16 (16.3%)	4 (18.2%)
Endometriosis	11 (11.2%)	2 (9.1%)
DM	7 (7.1%)	2 (9.1%)
Nulliparity	62 (63.2%)	14 (63.6%)
Recurrence time, month	19 (3–96)	12 (4–90)
Regimens after recurrence		
Hysterectomy	18 (18.4%)	12 (54.5%)
Progestin	46 (46.9%)	6 (27.3%)
GnRHa	34 (34.7%)	4 (18.2%)

BMI, body mass index; EC, endometrial carcinoma; AEH, atypical endometrial hyperplasia; PCOS, polycystic ovary syndrome; DM, diabetes mellitus.

Eighteen patients decided to receive hysterectomy directly after the first recurrence. According to the postoperative pathologic diagnosis, four of them were diagnosed as AEH, 12 were diagnosed as stage IA, one was stage IB, and one had co-occurrence of stage IC ovarian endometrial carcinoma. The remaining 80 patients still had the desire for fertility and underwent fertility-sparing re-treatment: 46 (46.9%) women were treated with progestin regimen and 34 (34.7%) with GnRHa-based therapy.

### Treatment Outcomes of the First Fertility-Sparing Re-Treatment

**Figure 1:**
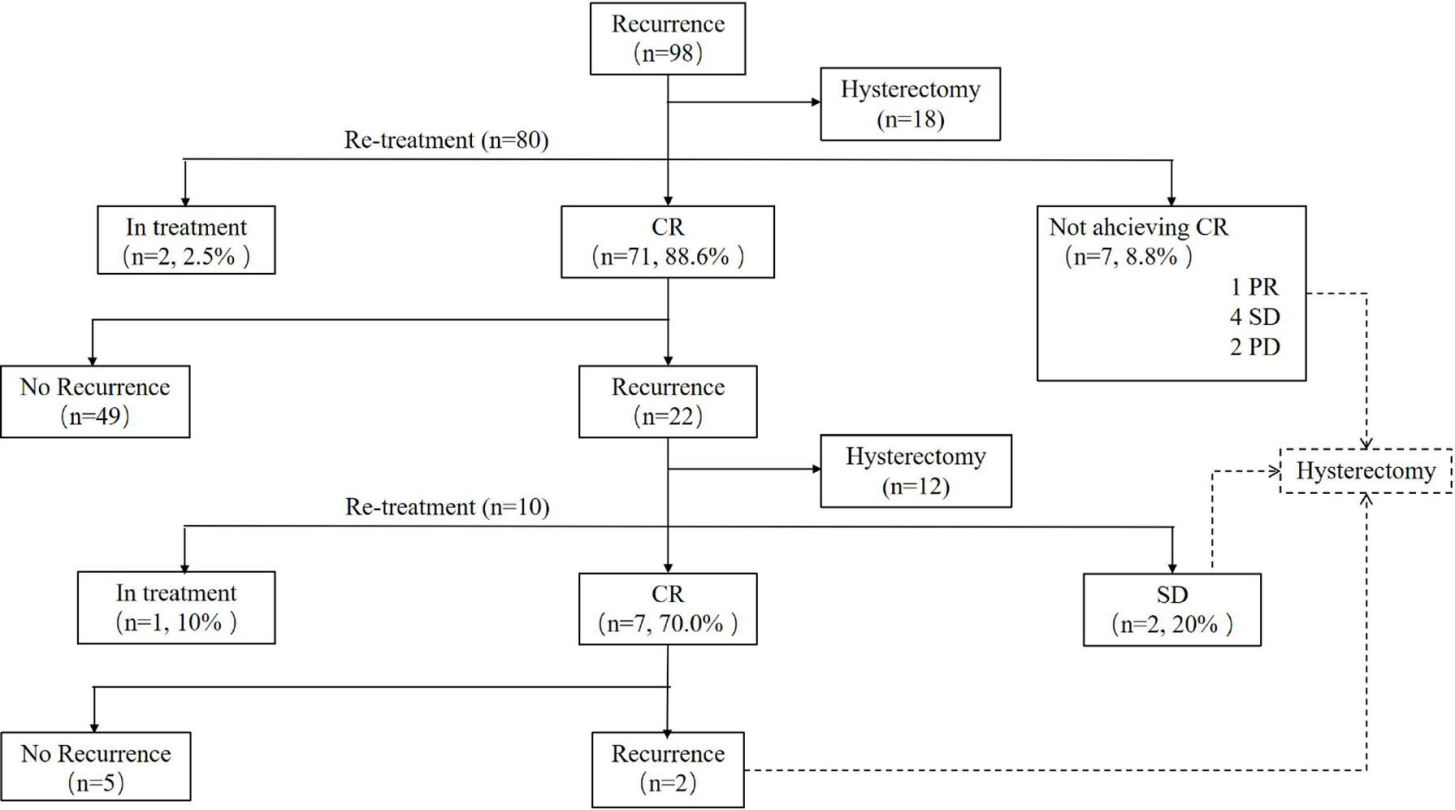
Outcomes of patients who received re-treatment. CR, complete response; PR, partial response; SD, stable disease; PD, progressive disease.

Of 80 patients who received repeated conservative treatment after relapse, 71 (88.6%) patients achieved CR with the 6 (3–16) months’ median CR time. Seven (8.8%) patients failed to achieve CR, one PR, four SD, and two PD and then underwent hysterectomy with or without lymphadenectomy (**Figure 1**). Based on pathological findings, two cases were diagnosed as AEH, three cases were stage IA EC, one case was stage IIIA EC, and one case was diagnosed as stage IIIB carcinosarcoma. The remaining two (2.5%) women were still in treatment. All patients were alive without tumors at the final contact.

The CR rate was 96.0% in AEH patients and 75.8% in EC patients (p = 0.233). The median time to CR was 6 months (3–16 months) in AEH patients and 6 months (3–11 months) in EC patients. High remission rates were found in patients who were younger than 35 years (91.1% *vs.* 85.7%, p = 0.452), who lost more than 10% of their own weight (100% *vs.* 89.2%, p = 0.623), who received progestin regimen (93.1% *vs.* 85.3%, p = 0.405), and whose BMI <28 (91.9% *vs.* 87.2%, p = 0.507) (**Table 2**).

**Table 2:** Predictors of complete response.

Predictors of complete response	OR (95% CI)	p-Value
Age: <35 *vs.* ≥35 years	1.708 (0.423–6.904)	0.452
Obesity: no *vs.* yes	1.667 (0.369–7.531)	0.507
AEH *vs.* EC	2.437 (0.565–2.437)	0.233
Weight loss: <10% *vs.* ≥10%	0.892 (0.824–0.966)	0.623
Regimen: progestin *vs.* GnRHa	1.810 (0.448–7.322)	0.405

EC, endometrial carcinoma; AEH, atypical endometrial hyperplasia; PCOS, polycystic ovary syndrome; GnRHa, gonadotropin-releasing hormone agonist.

**Figure 2:**
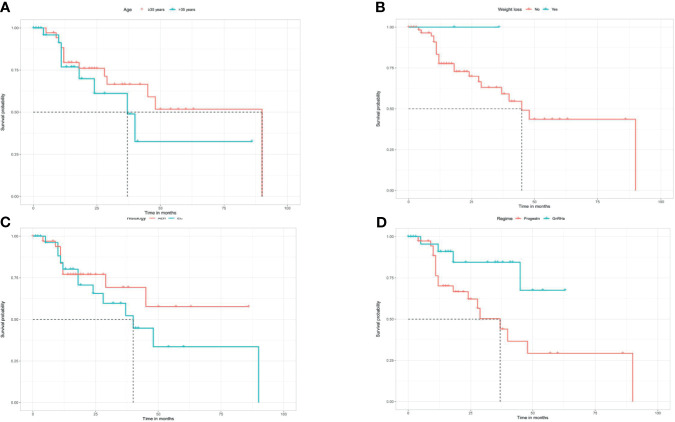
Kaplan Meier disease-free survival analysis for age **(A)**, weight loss **(B)**, histology **(C)**, and regimen **(D)**.

After a median follow-up time of 42 months (8–143 months), 22 (31.0%) women had developed recurrence again. The median time to the second recurrence was 12 months, ranging from 4 to 90 months. Patients’ characteristics of the second recurrence are summarized in **Table 1**. Patients who received GnRHa regimen, lose more than 10% weight, with AEH, or not obese had a low probability of recurrence (**Table 3**). The disease-free survival (DFS) of patients is shown in **Figure 2**.

**Table 3:** Predictors of recurrence.

Risk factors to recurrence	Univariate analysisHR (95% CI)	p-Value	Multivariate analysis HR (95% CI)	p-Value
Age: <35 *vs.* ≥35 years	0.923 (0.333–2.562)	0.878		
Obesity: no *vs.* yes	0.475 (0.160–1.412)	0.181		
AEH *vs.* EC	0.438 (0.157–0.223)	0.115		
Weight loss: <10% *vs.* ≥10%	0.959 (0.905–1.016)	0.371		
Regimen: progestin *vs.* GnRHa	4.687 (1.384–15.872)	**0.013**	3.643 (0.936–14.185)	0.062
Conceive: no *vs.* yes	1.624 (0.400–6.598)	0.498		

EC, endometrial carcinoma; AEH, atypical endometrial hyperplasia; PCOS, polycystic ovary syndrome; GnRHa, gonadotropin-releasing hormone agonist.

The bold value highlights the p value < 0.05.

At the second recurrence, 12 patients discontinued uterus preservation and chose to receive hysterectomy with or without lymphadenectomy. Extrauterine lesions were identified in two patients who received adjuvant therapy after surgery. Ten patients, three EC, and seven AEH decided to receive the third round of fertility-sparing treatment. Seven (70.0%) patients, 33.3% in EC and 85.7% in AEH, achieved CR again with the 6 (3–18) months’ median CR time (**Table 4**). Hysterectomy was performed in two (20.0%) patients due to SD. The remaining of one patient was still in treatment at the final contact.

**Table 4:** Outcome of first and second re-treatment of patients.

Characteristics	First re-treatment			Second re-treatment		
	EC (n = 29)	AEH (n = 51)	Total (n = 80)	EC (n = 3)	AEH (n = 7)	Total (n = 10)
CR						
CR rate	22 (75.8%)	49 (96.0%)	71 (88.7%)	1 (33.3%)	6 (85.7%)	7 (70.0%)
CR time, month (range)	6 (3–11)	6 (3–16)	6 (3–16)	6	4 (3–18)	6 (3–18)
Recurrence						
Recurrence rate	10 (45.5%)	12 (24.5%)	22 (31.0%)	1 (33.3%)	1 (14.3%)	2 (28.6%)
Recurrence time, month (range)	18 (5–90)	12 (4–48)	12 (4–90)	8	22	15 (8–22)

CR, complete response; EC, endometrial carcinoma; AEH, atypical endometrial hyperplasia.

For the seven CR women after the third round of fertility-preserving therapy, repeated recurrence occurred in two (28.6%) patients with the 15 months’ median recurrence time, and both of them received laparoscopic staging of EC, and both of them were diagnosed as stage IA EC according to postoperative histology. No patient died of the disease during this period (**Figure 1**).

### Adverse Effects

In progestin regimens, weight gain of more than 10% was the most common side effect (22.2%), followed by irregular bleeding (4.4%) and abnormal liver function (2.5%). In GnRHa regimens, irregular bleeding and postmenopausal symptoms such as hot flashes and vaginal dryness were the most common adverse effects (14.2%). The degree of menopause symptoms was minor, and no patients received add-back estrogen. No weight gain, liver dysfunction, intrauterine device (IUD) dislocation, or thromboembolism was recorded. The scheduled treatment was not delayed due to these minor side effects. No treatment-related deaths were identified.

### Fertility Outcomes

After the first fertility-sparing re-treatment, 49 women attempted to get pregnant, and 33 (67.3%) women were transferred to receive ART. Totally, 13 (26.5%) patients became pregnant, seven (14.3%) of them successfully delivered, and six (12.2%) of them miscarried (**Table 5**). Pregnancy rate was superior in patients who lose weight of more than 10% (100% *vs.* 29.6%, p = 0.021). Higher probability was observed in patients who were younger than 35 years (31.0% *vs.* 20.0%, p = 0.390). ART showed high tendency of pregnancy (30.2% *vs.* 0.0%, p = 0.116). After the third-round treatment, six women had the desire to conceive immediately; and all of them accepted ART, but no one became pregnant successfully.

**Table 5:** Pregnancy after re-treatment.

Characteristics	AEH	EC	Total
Attempts to conceive	36	13	49
Natural conception	9 (25.0%)	1 (7.7%)	16 (32.7%)
ART	27 (75.0%)	3 (23.1%)	33 (67.3%)
Total number of pregnancies	9 (25.0%)	4 (30.8%)	13 (26.5%)
Live baby delivery	6 (16.7%)	1 (7.7%)	7 (14.3%)
Miscarriage	3 (8.3%)	3 (23.1%)	6 (12.2%)

ART, assisted reproductive technology; EC, endometrial carcinoma; AEH, atypical endometrial hyperplasia.

## Discussion

With an increasing incidence of EC in younger women, increasingly more women are likely to seek conservative management options to preserve their uterus. Fertility-sparing management is well known and has been used for young women with EC and AEH who wish to preserve their fertility (12). Previous studies revealed a high remission rate of conservative treatment, as well as an association with a high rate of relapse, ranging from 10% to 88%, which is the most problematic feature of this therapy (13–18). No consensus has been reached on the treatment of recurrence after fertility preservation. Most patients underwent definitive surgical management including hysterectomy (19, 20), but some cases are still interested in childbearing after recurrence. The indications and protocol of repeated conservative treatment are rarely mentioned in previous literature. Therefore, it is a big challenge for the management of recurrence cases and selection of women for fertility-sparing re-treatment, which becomes increasingly complex. However, only a few studies have reported the outcomes of the second round of fertility-sparing management as part of their report until now (9, 11, 21, 22). The study aimed to investigate the efficacy and safety of fertility-preserving re-treatment for patients with recurrent EC or AEH who wish to preserve their fertility.

In our institution, patients who still want to preserve their fertility at recurrence should meet the criteria for initial conservative treatment; 81.6% of patients at the first recurrence and 45.5% at the second recurrence received fertility-sparing re-treatment, and the remaining underwent definitive surgical management including hysterectomy. The selection of regimen and dosage of drugs should be following the regimen in initial treatment, physicians’ recommendation, and patients’ choices.

The CR rate was 88.7% in second-round treatment and 70.0% in third-round treatment, and as the treatment times increased, the remission rate decreased. We speculate that the reason for the lower response rate in the re-treatment may be related to the pathological progression after recurrence and the insensitivity of imaging assessment of the superficial muscular involvement. In addition, a lower CR rate was noticed in EC group compared with AEH group, while there was no significant group difference, in accordance with former research (11, 14). In patients with AEH, the CR rate for re-treatment tended to be equal to that of the initial treatment. But in patients with EC, the CR rate after re-treatment tended to be lower than that of the initial treatment, which was similar to the results of retreated cases in other series (9, 17). The median CR time of re-treatment was 6 months as compared with the initial treatment according to previous studies (23). Considering the limited number of patients and some patients who are still under treatment, whose therapeutic efficacy is unable to assess, we hypothesize that a future study with larger samples may attain statistical significance.

About 31.0% of patients relapsed after the re-treatment, and 28.6% even had a third recurrence. The recurrence rate was not significantly higher than the initial treatment, while its probability increases continually with time. The median time of first and second recurrences was 19 and 15 months, respectively. We suggested that with the times of treatment increased, the recurrence time shortened. The time of the third recurrence was not significantly shortened because only two patients had a third recurrence. It appears necessary to follow a stricter follow-up regimen after the re-treatment than after the initial treatment to ensure that patients remain eligible for the treatment. The latest recurrence in our cohort took place at 90 months; others also report them at 13 years. They proposed that the risk of relapse is the highest at 1 year after a CR and then peaks again at the 7-year mark, and after this slowly decreases. Therefore, long-term monitoring and regular evaluation are of great importance.

It is not uncommon for young EC patients to have synchronous ovarian cancer. Some previous reports have suggested that in 2%–4% of patients, there is a risk of duplicated ovarian cancer or an increase in cancer stage (≥stage II), which was also observed in our research. Therefore, when intrauterine recurrence is identified, it is necessary to confirm whether the tumor is limited to the endometrium, and total evaluation was needed to ensure that the patient meets the eligibility criteria for the repeated treatments. Given the above, some scholars have suggested that EC patients who wanted to preserve fertility should be routinely performed a laparoscopy to exclude the possibility of synchronous ovarian tumors.

Analysis of possible response predictors singled out several factors: age, BMI, and weight control have been reported as factors predictive of remission and recurrence in previous reports. Age at diagnosis was a significant contributor to remission rate in women younger than 35 years as opposed to patients of 35 years of age and older (24), which was also found in our research. Besides, patients who were obese and lose weight <10% have a lower response and pregnancy rates, as well as higher recurrence rates, consistent with previous studies (25). Young patients with AEH/EC frequently have a history of obesity, which is usually associated with prolonged, unopposed estrogen exposure, accounting for the increased risk factor of EC in obese women (26, 27). Comorbidities such as PCOS and DM did not affect the oncologic outcome of conservative treatment, which is consistent with former studies (28, 29). But patients were unable to conceive due to obesity and PCOS, leading to anovulation; and the absence of stimulation of progestin may also increase the risk of recurrence (30). Herein, weight control and health consulting are crucial in the whole lifespan management of fertility-sparing treatment. Another study supported that longer menstrual cycles and infrequent menstrual bleeding appear as independent predictive factors for conservative treatment failure (31). These risk factors could help us to identify responsive women as well as women who are candidates for a stricter follow-up before treatment. In other words, for recurrent patients with high-risk factors, such as those older than 35 years and obese, fertility-sparing re-treatment should be carefully performed, complete assessment and close monitoring should be performed.

Usually, the re-treatment regimen was high-dose oral progestin. Other methods such as LNG-IUS, GnRHa, letrozole, and metformin have been reported as options for preserving women’s fertility with EC and AEH and proved to have an encouraging result (32). In our series, 93.1% in regimen progestin and 85.3% in regimen GnRHa achieved CR; both progestin and GnRHa combined regimens had a great response rate. But high recurrence rate was found in regimen progestin. Also, GnRHa combined therapy has an advantage on weight control compared with progestin therapy since we all know that weight gain was the main side effect of high-dose progestin. Herein, GnRHa combined regimen could be an alternative option for recurrence patients who were unsuitable for progestin, such as obesity and abnormal liver function.

Heterogeneity of tumor was observed in our study. Two recurrent patients received hysterectomy, and the postoperative pathology was carcinosarcoma with the endometrial adenocarcinoma initial diagnosis. Also, another patient’s immunohistochemistry (IHC) status changed at the time of recurrence. At the primary treatment, the patient’s IHC showed mismatch repair (MMR)-proficient (pMMR), but after recurrence, the IHC was MMR-deficient (dMMR), and her gene test proved to be Lynch syndrome. This patient had co-occurrence of bone metastasis at recurrence and underwent chemotherapy and radiotherapy. Due to the data limitation, we failed to find the biomarker and the possible reason for treatment failure. And the association between MMR and response is unclear (33). Some articles have proposed that the overall and recurrence-free survival was significantly lower in p53 abnormal and dMMR patients subgroups and that MMR deficiency appears as a highly specific predictor of recurrence of AEH/EC after initial regression (34). Thus, patients with Lynch syndrome and p53 mutations may not be treated conservatively (35). Also, we found a decrease of progesterone receptor (PR) in one patient. With the time of therapy prolonged, the receptor of progesterone decreased from 90% to negative. We change the regimen from progestin to GnRHa in this patient when we notice this phenomenon, and she finally achieved CR after three courses. The status of PR and estrogen receptor (ER) was thought to be associated with disease regression in some research, and the weak expression of PR-B could be a predictive factor of no response and recurrence (36–38), while others indicated that many ER- and PR-negative lesions would still respond to hormonal therapy, but they are against routine ER and PR tissue expression testing, as this does not change the management plan (24, 28). But we believe that GnRHa combined therapy might be an option for the patient who is PR negative instead of progestin. Other markers such as PTEN, HE4, and PAX-2 were also investigated, but these factors seem not to be useful as predictive markers of response to the conservative treatment (38, 39). As the modern The Cancer Genome Atlas (TCGA)-based molecular classification system has been validated, it might help to predict the response and contribute to the selection of population who are suitable for fertility-preserving treatment.

We believe that the achievement of pregnancy is probably the most important indicator of the success of uterine preservation. The ultimate goal of fertility-sparing management of AEH and EC is to obtain live birth. According to reports in the literature, the pregnancy rate after CR is about 30% (40–42). In our study, after second-round fertility-sparing management, 26.5% of patients became pregnant, 14.3% of them successfully delivered, and 12.2% of them miscarried. And all the pregnancies occurred under the assistance of ART. The above results indicated that patients can achieve pregnancy after being re-treated for recurrent disease. What is more, once CR has been achieved, pregnancy should be carried out as soon as possible, and ART is recommended without causing significant delays. But after the third-round treatment, no patients got pregnant even ART was performed. This might be due to the repeated hysteroscopic evaluation and curettage, which causes damage to the endometrium. So before patients undergo treatment, they should be informed that even though the uterus could be preserved, the possibility of conception was still low especially in the second recurrence.

### Limitations

It is one of the largest studies that focused on repeated fertility-sparing management in patients with recurrent AEH and EC. But the present study has a few limitations that should be discussed. First, this was a single-center retrospective study. The treatment outcomes in the present study may differ from those in previous studies. Second, in some cases, it was not possible to collect all clinical data, as we only used the medical records. Third, some patients were still under treatment until the last follow-up, which may influence the results of the research.

## Conclusions

In conclusion, based on the abovementioned data, fertility-sparing re-treatment appears to be an acceptable treatment option for recurrent EC and AEH patients with high rate of regression and minor side effects. Besides, it is feasible and can allow young patients to conceive even when re-treatment applied. But repeated recurrence still happened, and a stricter follow-up regimen after re-treatment is needed.

## Data Availability Statement

The raw data supporting the conclusions of this article will be made available by the authors, without undue reservation.

## Author Contributions

JC, JY, DC, and KS: conceived and designed the study. JC, MY, HZ, JW, YZ, NC, and PP: data acquisition. JC and DC: analyzed the data. JC: wrote the original draft. JY, DC, and KS: wrote, reviewed, and edited. All authors contributed to the article and approved the submitted version.

## Funding

Fund program: National Natural Science Foundation of China (81101975).

## Conflict of Interest

The authors declare that the research was conducted in the absence of any commercial or financial relationships that could be construed as a potential conflict of interest.

## Publisher’s Note

All claims expressed in this article are solely those of the authors and do not necessarily represent those of their affiliated organizations, or those of the publisher, the editors and the reviewers. Any product that may be evaluated in this article, or claim that may be made by its manufacturer, is not guaranteed or endorsed by the publisher.
